# Tinea versicolour in underrepresented groups: An All of Us database analysis

**DOI:** 10.1002/ski2.152

**Published:** 2022-07-21

**Authors:** Isabelle Moseley, Sara D. Ragi, Samantha Ouellette, Babar Rao

**Affiliations:** ^1^ The Warren Alpert Medical School of Brown University Providence RI USA; ^2^ Department of Dermatology Robert Wood Johnson Medical School New Brunswick NJ USA

## Abstract

Tinea versicolour, used interchangeably with pityriasis versicolour (PV), is a superficial fungal infection of the stratum corneum caused by Malassezia furfur, a fungus of the normal flora of the skin. PV occurs when conditions favour proliferation of the organism's mycelial form, such as in environments with high temperatures/humidity, in immunodeficient/immunocompromised states, and during pregnancy. PV presents as numerous well‐ demarcated macules with a powdery scale. Prior epidemiologic studies have indicated that underrepresented groups defined by race experience a higher burden of PV as compared to White patients. However, the burden of PV in other underrepresented groups has not previously been examined, as underrepresented groups are frequently excluded from studies evaluating the impact of dermatologic disease. The new National Institute of Health All of Us Research Program (AoU) aims to build one of the world's largest and most diverse databases to promote elucidation of health disparities, particularly in communities that have been historically excluded from biomedical research.

**Key points**

- The novel National Institute of Health All of Us Research Program (AoU) includes a ethnically/racially diverse patient database particularly including communities that have been traditionally excluded from biomedical research.
- We found that underrepresented groups in the AoU database including Black patients, Hispanic patients, and participants with physical disabilities may possess an increased risk of pityriasis versicolour.
- Uninsured patients, patients with less than a high school degree, and patients with a household income ≤$35,000 may have limited access to dermatologic care for PV and experience underdiagnosis may be occurring in these patient populations.

Dear Editor, Tinea versicolour, used interchangeably with pityriasis versicolour (PV), is a superficial fungal infection of the stratum corneum caused by Malassezia furfur, a fungus of the normal flora of the skin.^1^ PV occurs when conditions favour proliferation of the organism's mycelial form, such as in environments with high temperatures/humidity, in immunodeficient/immunocompromised states, and during pregnancy.^1^ PV presents as numerous well‐ demarcated macules with a powdery scale.^2^ Prior epidemiologic studies have indicated that underrepresented groups defined by race experience a higher burden of PV as compared to White patients.^2^, ^3^ However, the burden of PV in other underrepresented groups has not previously been examined, as underrepresented groups are frequently excluded from studies evaluating the impact of dermatologic disease.^4^ The new National Institute of Health All of Us Research Program (AoU) aims to build one of the world's largest and most diverse databases to promote elucidation of health disparities, particularly in communities that have been historically excluded from biomedical research.^4^ AoU defines underrepresented groups prioritized for analysis based not only on race/ethnicity but also age (≥75 years), disability (inability to perform everyday physical activities), sexual orientation/gender identity (lesbian, gay, bisexual, transgender, queer, intersex, and asexual (LGBTQIA+), income (annual household income ≤$35,000), and education (less than a high school degree).^4^ We used the latest AoU data release to evaluate the disease burden of PV among underrepresented groups defined by this novel framework. Here we found that underrepresented groups in the AoU database including Black patients, Hispanic patients, and participants with physical disabilities demonstrated possessed increased risk of pityriasis versicolour, which is consistent with the literature. Additionally, we found that uninsured patients, patients with less than a high school degree, and participants with a household income ≤$35,000 may have limited access to dermatologic care for PV and underdiagnosis in these patient populations.

We linked survey and electronic health record data to estimate the prevalence of PV upon enrolment in each demographic group. The AoU database includes information about participants over the age of 18 who signed up directly through the AoU website or are a part of a participating health care provider organization. We evaluated AoU Registered Tier dataset version 5, which includes data collected between 30 May 2017 and 1 April 2021. AoU v5 includes 329,038 participants. Of these, 251,597 (76.5%) had Electronic Health Record data and 1698 had PV (overall prevalence, 0.67%; 95% confidence interval (CI), 0.64–0.71) (Table 1). We used multivariate logistic regression adjusted for the aforementioned variables in addition to health insurance status and immunodeficiency to estimate the adjusted odds ratio (OR) for a PV diagnosis in each underrepresented group. Compared to White participants, Black and Hispanic participants had a higher adjusted odds of PV (OR, 1.38; 95% CI, 1.21–1.59 and OR, 1.26; 95% CI, 1.09–1.44, respectively) (Table 1). LGBTQIA+ participants had a nonsignificant increased aOR (OR, 1.09; 95% CI, 0.93–1.05) and physically disabled participants had a significantly increased aOR (OR, 1.26; 95% CI, 1.07–1.47). Lower adjusted odds of PV diagnosis were observed in participants with age ≥75 (OR, 0.49; 95% CI, 0.37–0.63), less than a high school degree (OR, 0.80; 95% CI, 0.65–0.97), household income ≤$35,000 (OR, 0.78; 95% CI, 0.68–0.90), and no health insurance (OR, 0.42; 95% CI, 0.31–0.56).

**TABLE 1:** Prevalence of tinea versicolour in underrepresented groups enroled in the All of Us Research Program

	All enroled, *n* (%)	EHR data, *n* (%)	Pityriasis versicolour, *n* (%)	Age	sd	Prevalence estimate (95% CI)	Univariate regression OR (95% CI)	Multivariate regression OR (95% CI)^d^
Race/Ethnicity								
White	172753 (52.50)	138793 (55.16)	869 (51.18)	55.18	16.74	0.039 (0.038–0.040)	Ref	Ref
Black	67897 (20.64)	47564 (18.90)	357 (21.02)	50.15	14.48	0.114 (0.111–0.116)	1.14 (1.00–1.29)	1.38 (1.21–1.59)
Hispanic	60535 (18.40)	45195 (17.96)	335 (19.73)	45.07	15.72	0.120 (0.117–0.123)	1.05 (0.93–1.20)	1.26 (1.09–1.44)
Other^a^	21726 (6.60)	15640 (6.22)	110 (6.48)	44.88	16.85	0.346 (0.338–0.353)	1.00 (0.81–1.21)	0.99 (0.81–1.21)
Age								
<75	309632 (94.11)	235381 (93.57)	233741 (93.55)	49.88	15.55	0.023 (0.022–0.024)	Ref	Ref
≥75	19375 (5.89)	16186 (6.43)	16128 (6.45)	79.37	2.87	0.334 (0.327–0.341)	0.51 (0.39–0.66)	0.49 (0.37–0.63)
Gender								
M	124196 (37.75)	92855 (36.91)	716 (42.17)	54.14	16.54	0.058 (0.057–0.060)	Ref	Ref
F	197949 (60.17)	153790 (61.13)	948 (55.83)	50.48	16.60	0.035 (0.034–0.036)	0.76 (0.69–0.84)	0.69 (0.63–0.76)
Other	6862 (2.09)	4922 (1.96)	34 (2.00)	47.93	17.71	1.098 (NaN)	0.82 (0.57–1.14)	0.81 (0.56–1.14)
LGBTQIA+ status								
N	290158 (88.19)	222953 (88.63)	1477 (86.98)	52.54	16.57	0.024 (0.024–0.025)	Ref	Ref
Y	38849 (11.81)	28614 (11.37)	221 (13.02)	45.86	16.59	0.189 (0.184–0.193)	1.08 (0.93–1.24)	1.09 (0.93–1.25)
Education								
Completed college	140529 (42.71)	112271 (44.63)	819 (48.23)	53.64	16.96	0.048 (0.047–0.049)	Ref	Ref
Completed high school	148725 (45.20)	111279 (44.23)	708 (41.70)	50.10	16.69	0.049 (0.047–0.050)	0.83 (0.75–0.92)	0.84 (0.75–0.93)
Less than high school	32177 (9.78)	22874 (9.09)	136 (8.01)	50.44	14.79	0.236 (0.231–0.242)	0.79 (0.65–0.94)	0.80 (0.65–0.97)
Income^b^								
>35K	191368 (58.17)	151178 (60.09)	1078 (63.49)	53.31	16.56	0.036 (0.035–0.037)	Ref	Ref
≤35k	73942 (22.47)	53371 (21.22)	312 (18.37)	47.50	15.98	0.101 (0.099–0.104)	0.76 (0.67–0.86)	0.78 (0.68–0.90)
Health insurance								
Yes	296627 (90.16)	230731 (91.72)	1621 (95.47)	52.37	16.79	0.023 (0.023–0.024)	Ref	Ref
No	23235 (7.06)	14921 (5.93)	47 (2.77)	44.51	13.03	0.362 (0.355–0.370)	0.41 (0.30–0.54)	0.42 (0.31–0.56)
Disability^c^								
w/o Disability	284833 (86.59)	223287 (88.76)	1481 (87.22)	51.55	16.99	0.024 (0.024–0.025)	Ref	Ref
w/disability	31413 (9.55)	24551 (9.76)	191 (11.25)	53.64	13.79	0.220 (0.215–0.225)	1.21 (1.04–1.41)	1.26 (1.07–1.47)

*Note*: Columns for risk factors present the prevalence of each risk factor in each subgroup.

Abbreviations: BMI, body mass index; EHR, electronic health record; LGBTQIA+, lesbian, gay, bisexual, transgender, queer, intersex, and asexual.

^a^ The “other” category comprises the following categories from All of Us questionnaires: Another single population: participants self‐reporting either Middle Eastern or North African or Native Hawaiian or other Pacific Islander (please note All of Us does not provide disaggregated data on these yet). None of these populations: participants self‐reporting “None of these fully describe me” (options are White, Black, African American, or African, Asian, Middle Eastern or North African, Native Hawaiian or other Pacific Islander). > 1, non‐Hispanic >1 race selected.

^b^ Income corresponds to annual household income.

^c^ Disability indicates physical disability (participants who answered that they cannot carry out every day physical activities at all or only a little).

^d^ Multivariate model adjusts for race, ethnicity, age, sex, household income, education, physical disability, and health insurance status.

This study provides important evidence to confirm the scientific consistency of the AoU database, indicating that it is a valuable resource for evaluating the burden of dermatologic disease in underrepresented groups. The prevalence of PV in AoU is consistent with the approximate 1% prevalence reported in numerous prior epidemiologic studies.^1^, ^2^, ^3^ Among underrepresented groups in AoU, Black patients, Hispanic patients, and participants with physical disabilities had increased odds of pityriasis versicolour, which is also consistent with prior findings.^2^ Black patients have previously been reported to have the highest rate of dermatology visits for PV which may be explained by an increased susceptibility, or differences in health care utilization including the use of medical services for PV.^2^, ^5^ PV may exhibit a greater disease burden in patient populations with pigmented/darker skin.^2^, ^5^ The decreased odds of PV diagnosis in patients without health insurance, those with less than a high school degree, and participants with household income ≤$35,000 may indicate limited access to dermatologic care and underdiagnosed PV in these populations, leading to increased morbidity and decreased health‐related quality of life. In conclusion, the AoU research program provides a useful platform for evaluating the burden of dermatologic diseases among underrepresented groups.

## CONFLICTS OF INTEREST

None to declare.

## AUTHOR CONTRIBUTIONS

**Isabelle Moseley**: Conceptualization (Equal); Data curation (Equal); Formal analysis (Equal); Methodology (Equal); Writing – original draft (Equal). **Sara D. Ragi**: Project administration (Equal); Writing – review & editing (Equal). **Samantha Ouellette**: Writing – review & editing (Equal). **Barbar Rao**: Supervision (Equal); Validation (Equal); Writing – review & editing (Equal).

## Data Availability

The data that support the findings of this study are openly available at https://allofus.nih.gov/.
